# Phosphorylation of FE65 at threonine 579 by GSK3β stimulates amyloid precursor protein processing

**DOI:** 10.1038/s41598-017-12334-2

**Published:** 2017-09-29

**Authors:** Yat Shing Lee, Wan Ning Vanessa Chow, Kwok-Fai Lau

**Affiliations:** School of Life Sciences, Faculty of Science, The Chinese University of Hong Kong, Shatin, N.T., Hong Kong SAR

## Abstract

Excessive generation of amyloid-β peptide (Aβ) by aberrant proteolysis of amyloid precursor protein (APP) is a key event in Alzheimer's disease (AD) pathogenesis. FE65 is a brain-enriched phospho-adaptor protein that interacts with APP and has been shown to modulate APP processing. However, the mechanism(s) that FE65 alters APP processing is still not fully understood. In the present study, we demonstrate that FE65 is phosphorylated at threonine 579 (T579) by glycogen synthase kinase 3β (GSK3β). Moreover, FE65 T579 phosphorylation potentiates γ- and β-secretases-mediated APP processing and Aβ liberation. Additionally, the phosphorylation suppresses FE65 PTB2 intermolecular dimerization but enhances FE65/APP complex formation. Hence, our findings reveal a novel mechanism that GSK3β stimulates amyloidogenic processing of APP by phosphorylation of FE65 at T579.

## Introduction

Deposition of extracellular amyloid-β (Aβ) plaques is a pathological hallmark of Alzheimer's disease (AD). Aβ is a 40–42 amino acid toxic peptide derived from regulated stepwise cleavage of amyloid precursor protein (APP) by β- and γ-secretase in the amyloidogenic pathway. Emerging evidence reveals that the proteolytic cleavage of APP can be influenced by the APP intracellular domain (AICD) interacting proteins including FE65^1,2^.

FE65, also known as APP-binding family B member 1 (APBB1), is a brain-enriched adaptor protein implicated in AD. FE65 exhibits abundant expression in hippocampus, the region in which AD patients is severely affected. FE65 possesses three conserved protein interaction domains, including an N-terminal WW domain and two C-terminal phosphotyrosine binding (PTB) domains, and is capable of recruiting different proteins to form multimolecular complexes. Of note, FE65 physically binds to AICD through its second PTB domain (PTB2), and serves as a bridging molecule between APP and a number of ApoE receptors including low-density lipoprotein receptor-related protein (LRP1), apolipoprotein E receptor 2 (ApoER2) and very-low-density-lipoprotein receptor (VLDLR) to modulate APP processing. Furthermore, AICD/ FE65 together with histone acetyltransferase Tip60 are capable of forming a transcriptionally active multimeric complex to regulate the transcription of a number of genes such as the β-secretase BACE1, the Aβ degrading enzymes neprylisin and the insulin-degrading enzyme genes which may in turn influence Aβ level^2,3^.

Despite the importance of FE65 in modulation of APP processing and AICD-FE65-mediated transcription, the underlying regulatory mechanism is not fully characterized. Protein phosphorylation is a common post-translational modification in cells that serves as a molecular switch for many signaling pathways. As such, a number of phosphorylation sites on FE65 implicated in different cellular processes have been reported^4–6^. It is therefore tempting to speculate that other FE65 phosphorylation sites are responsible for conferring an additional level of regulation to FE65 functions in relations to AD. Previously, we have identified several FE65 phosphorylation sites by mass spectrometry (^6^ and our unpublished data) including FE65 T579. Since T579 is located within the FE65 PTB2 domain which is for binding to APP, we speculated that FE65 T579 phosphorylation may alter APP metabolism, and attempted to identify the responsible kinase(s). By using various *in silico* kinase prediction tools including Group-based Prediction System Version 3.0 and NetworKIN 3.0^7,8^, glycogen synthase kinase 3β (GSK3β) was predicted to be a putative FE65 T579 kinase. In this study, we showed that FE65 is phosphorylated at T579 by GSK3β. Moreover, we demonstrated that FE65 T579 phosphorylation promotes FE65-mediated APP processing and Aβ liberation by promoting FE65/APP interaction through suppressing the FE65 PTB2 dimerization.

## Results

### GSK3β phosphorylates FE65 T579

**Figure 1:**
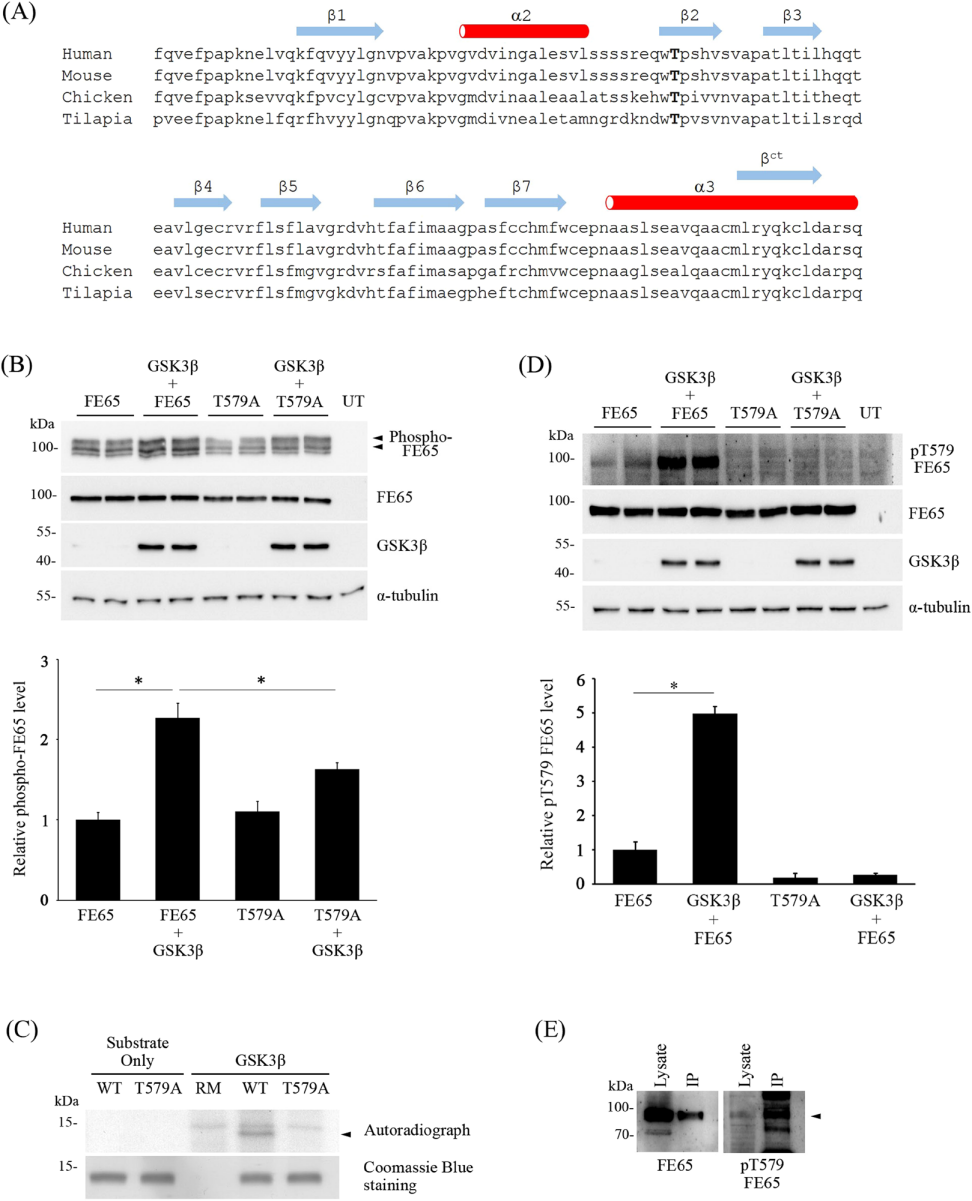
GSK3β phosphorylates FE65 T579 (**A**) Alignment of FE65 PTB2 sequences from various species. T597 is bolded (reference to human). Secondary structures of human FE65 PTB2 are indicated according to^18,19^. The position of β^ct^ is also shown. (**B**) FE65 and FE65 T579A were transfected to HEK293 cells together with or without GSK3β. Cell lysates were resolved on 8% (top panel) and 12% (second panel) SDS-PAGE gels for determination of FE65 migration patterns and total FE65 levels, respectively. Anti-FE65 E20 antibody was used to detect FE65. Expression of HA-tagged GSK3β was confirmed by immunoblotting using anti-HA 12CA5 antibody. α-tubulin was detected by anti-α-tubulin DM1A antibody as loading control. UT, untransfected. The relative amount of phosphorylated-FE65 was analyzed by densitometry (LI-COR® Image Studio™ Software). n = 4. *P < 0.001. Results are means ± S.D. (**C**) Bacterially expressed His-tagged FE65 PTB2 (531–666) wild-type/T579A were incubated with HA-tagged GSK3β immunoprecipitated from transfected cell lysate together with [γ-^32^P]-ATP for an hour at 30 °C. Arrow denotes the specific phosphorylated band in lane 4. Lower panel shows the Coomassie Blue staining. RM: reaction mixture only without substrate. WT, His-tagged FE65 PTB2 (531–666) wild-type. T579A, His-tagged FE65 PTB2 (531–666) T579A mutant. (**D**) FE65 and FE65 T579A were transfected to HEK293 cells together with or without GSK3β. T579 phosphorylated FE65 was detected by pT579 FE65 phospho-specific antibody. UT indicates untransfected. FE65, GSK3β and α-tubulin were detected by immunoblotting. The relative amount of p579 FE65 level was analyzed by densitometry. n = 6. *P < 0.001. Results are means ± S.D. (**E**) Endogenous FE65 was immunoprecipitated from adult rat brain lysate by using anti-FE65 E20. FE65 in the brain lysate and immunoprecipitate were revealed by immunoblotting. T579 phosphorylated FE65 was detected with pT579 FE65 antibody. Arrow denotes T579 phosphorylated FE65.

According to our unpublished mass spectrometric data, FE65 T579 within the PTB2 domain is a phosphorylated residue. Noteworthy, the residue is highly conserved among various vertebrates from mammal to fish which might suggest the biological significance of FE65 T579 (Fig. 1A). However, the kinase(s) that target the residue remains unknown. As FE65 T579 is followed by a proline, T579 might be a potential target for proline-directed protein kinases. Moreover, our *in silico* analysis by using Group-based Prediction System Version 3.0 and NetworKIN 3.0 indicates that GSK3β is a putative kinase for FE65 T579 (data not shown). To obtain experimental evidence, we investigated if GSK3β phosphorylates FE65 in cells. Since reduced electrophoretic mobility is a characteristic of many phospho-proteins including FE65^6^, we therefore monitored the band patterns and total amounts of FE65 from transfected cells by using 8% and 12% SDS-PAGE, respectively. As reported previously, three major bands of FE65 could be differentiated in an 8% SDS-PAGE gel (Fig. 1B, top panel), and the upper two species have been shown to be the phosphorylated FE65^6^. Intriguingly, the intensities of the two upper bands of FE65 were increased upon co-transfection with GSK3β. However, only a small increase in the upper bands were observed in FE65 T579A + GSK3β (i.e. FE65 + GSK3β vs T579A + GSK3β). As shown in the graph, the relative amount of phosphorylated FE65 was increased by 2.3-fold while FE65 T579A was only increased by 1.4-fold in the presence of GSK3β. Since T579A mutation could preclude phosphorylation of the residue, our finding suggests that overexpression of GSK3β leads to increased FE65 phosphorylation preferentially at T579.

Next, we tested if GSK3β phosphorylates FE65 T579 directly by *in vitro* kinase assay. GSK3β was immunoprecipitated from transfected cell lysate and incubated with bacterially expressed His-tagged FE65 PTB2 wild-type (WT) or T579A, together with [γ-^32^P]-ATP for 30 mins at 30 °C. The reaction mixtures were then resolved on 15% SDS-PAGE and exposed to an autoradiogram. As shown in Fig. 1C, GSK3β induced phosphorylation on His-tagged FE65 PTB2 WT (denoted with an arrow) but not on T579A mutant.

To further investigate whether GSK3β phosphorylates FE65 T579 in cells, we developed a phospho-specific FE65 T579 (pT579 FE65) antibody. The antibody was used for immunoblot analysis of the cell transfected with FE65, FE65 T579A, FE65 + GSK3β and FE65 T579A + GSK3β. As shown in Fig. 1D, pT579 FE65 antibody could detect a faint band from FE65 transfected cell lysates which could be a result of phosphorylation of FE65 by endogenous kinases including GSK3β. Remarkably, a strong band was observed from FE65 + GSK3β co-transfected cell lysates. Conversely, FE65 T579 phosphorylation was not detected in both FE65 T579A and FE65 T579A + GSK3β transfected cells as T579A mutation could preclude phosphorylation of the residue. Additionally, pT579 FE65 antibody could detect phosphorylated FE65 from rat brain immunoprecipitate (Fig. 1E). A weak pT579 FE65 band was also observed in rat brain lysate (Fig. 1E). Together, our findings reveal that FE65 T579 is a phosphorylated residue which can be targeted by GSK3β.

### GSK3β enhances FE65-mediated APP processing

**Figure 2:**
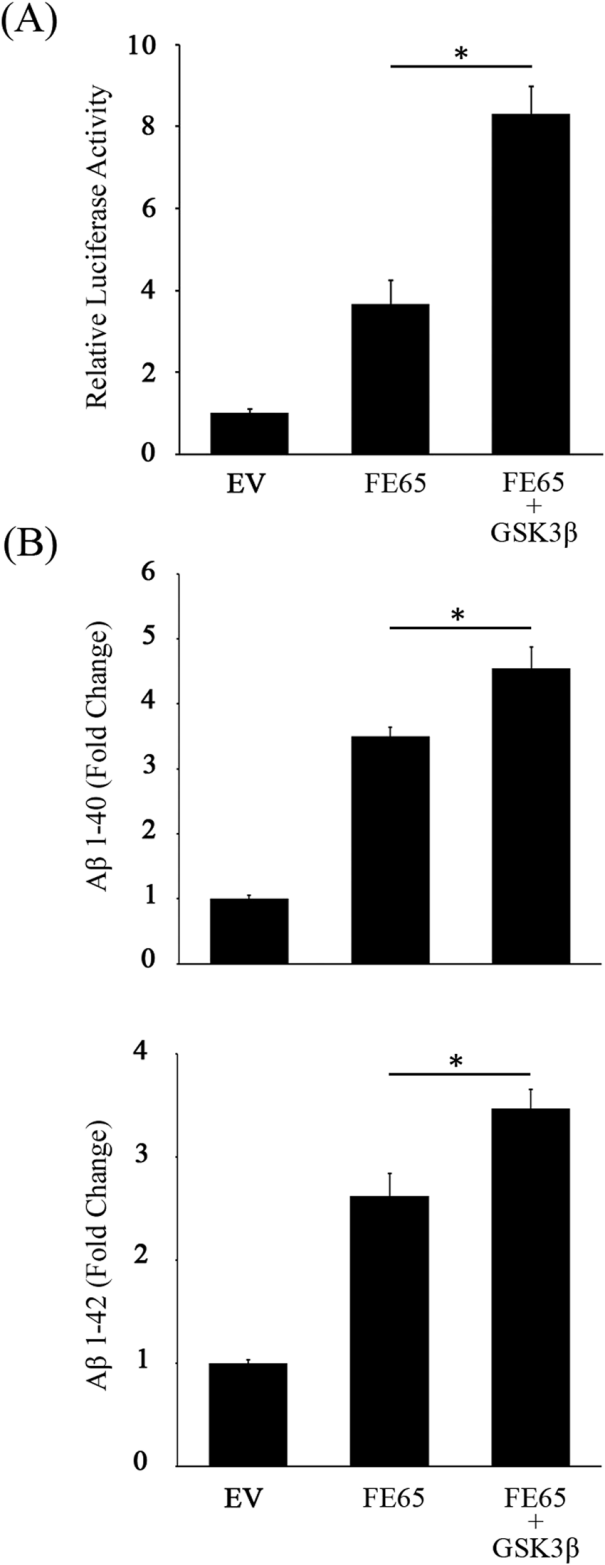
GSK3β enhances FE65-mediated APP processing. (**A**) HEK293 cells were co-transfected with GAL4-APP, pFR-Luc and phRL-TK together with empty vector (EV), FE65 or FE65 + GSK3β. GSK3β potentiates the stimulatory effect of FE65 on APP-GAL4 cleavage. n = 5. *P < 0.001. Results are means ± S.D. (**B**) HEK293 cells were co-transfected with APP + FE65 with or without GSK3β. Levels of secreted Aβ1–40 (top graph) and 1–42 (bottom graph) were measured by corresponding Aβ ELISA kits. Overexpression of GSK3β enhances the effect of FE65 on both Aβ1–40 and 1–42 liberation. n = 5. *P < 0.001. Results are means ± S.D.

Phosphorylation of FE65 has been shown to influence various FE65-mediated cellular processes including APP processing^5,9–11^. As we have found that GSK3β phosphorylates FE65 T579 within its APP binding PTB2 domain, we therefore enquired if GSK3β modulates FE65-mediated APP processing. In this regard, we employed a APP-GAL4 cleavage reporter system to determine the effect of GSK3β on FE65-mediated γ-secretase cleavage of APP^9,12^. Akin to previous report^13^, a significant increase in γ-secretase-mediated APP cleavage was observed when FE65 was overexpressed. Remarkably, the stimulatory effect of FE65 on γ-secretase cleavage of APP was further enhanced by 2.3-fold when co-transfected with GSK3β (Fig. 2A).

Aβ1–40 and 1–42 are two major components of the amyloid plaques derived from the amyloidogenic processing of APP. We therefore also monitored the effect of GSK3β on FE65-mediated Aβ1–40 and 1–42 liberation by ELISA assays. Consistent with previous reports^9,14^, both Aβ1–40 and 1–42 levels were elevated in cells overexpressing FE65. Such stimulatory effect of FE65 was further potentiated by co-transfection of GSK3β (Fig. 2B). Together, FE65-mediated APP processing and Aβ production are potentiated by GSK3β.

### FE65 T579 phosphorylation potentiates FE65-mediated APP processing

**Figure 3:**
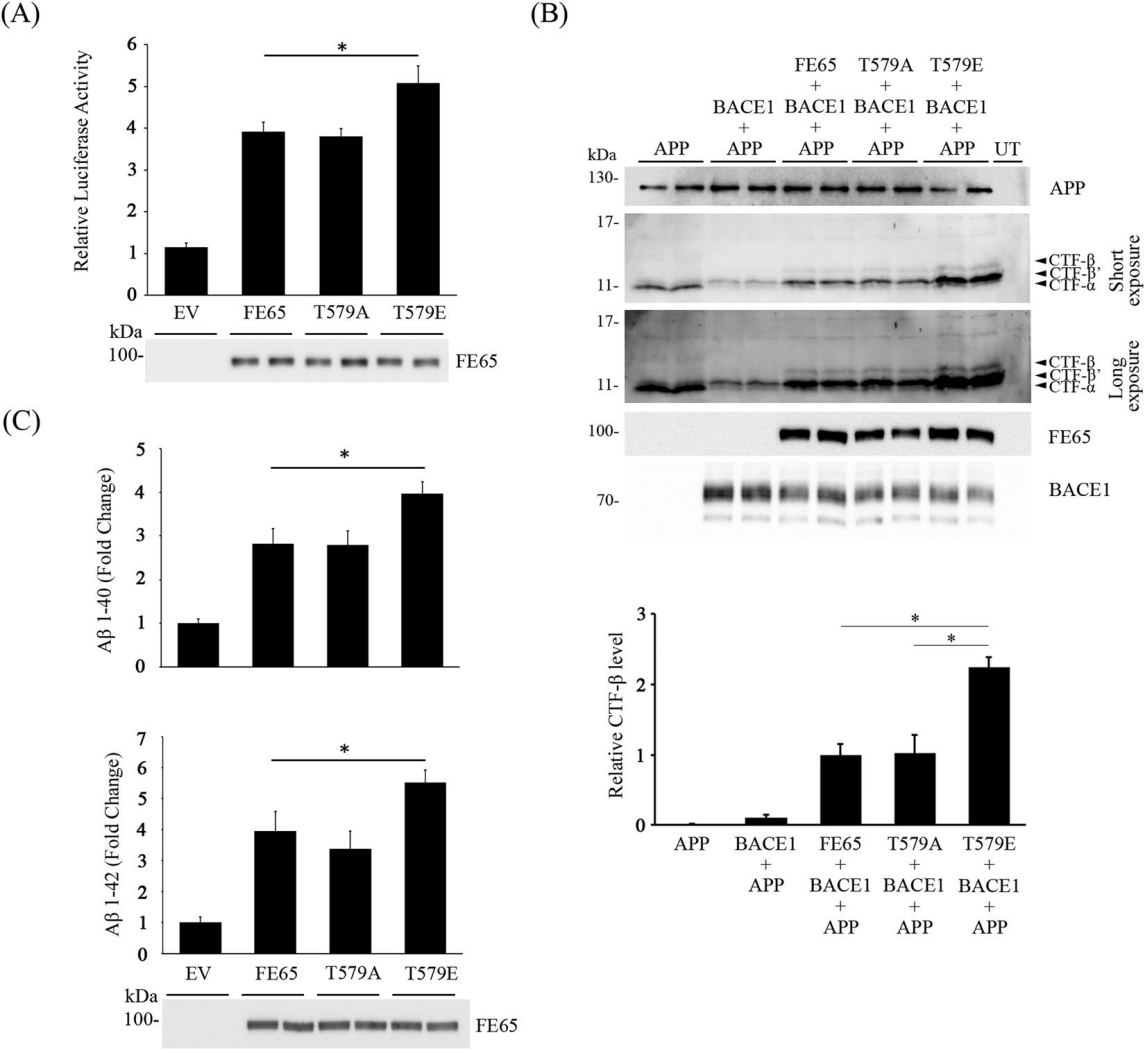
FE65 T579 phosphorylation potentiates FE65-mediated APP processing. (**A**) HEK293 cells were co-transfected with GAL4-APP, pFR-Luc and phRL-TK together with EV, FE65, FE65 T579A or FE65 T579E. Phosphomimetic mutation of FE65 T579 further enhances the effect of FE65 on APP-GAL4 cleavage. n = 5. *P < 0.001. Results are means ± S.D. The expression of FE65 and the mutants were determined by immunoblotting. (**B**) Cells were transfected with APP + BACE1 + FE65/T579A/T579E. Transfected cell lysates were resolved on 16% Tris-Tricine gel, and immunoblotted for APP CTFs. Arrows denote CTF-α, -β or -β’. UT: untransfected. Two exposures of CTF blot were shown (**C**) Cells were transfected with APP together with EV, FE65, FE65 T579A or FE65 T579E. Levels of secreted Aβ1–40 (top graph) and 1–42 (bottom graph) were measured by corresponding Aβ ELISA kits. FE65 T579E phosphomimetic mutation potentiates FE65-mediated Aβ liberation. n = 5. *P < 0.001. Results are means ± S.D.

As shown above, FE65-mediated APP processing is enhanced in the presence of GSK3β. Since the kinase is found to phosphorylate FE65 T579, we anticipated that FE65 T579 phosphorylation plays a role in regulating FE65-mediated APP processing. To further understand the effect of FE65 T579 phosphorylation, phosphomimetic approach was employed. FE65 T579 was mutated into glutamic acid (T579E) and alanine (T579A) to mimic permanent phosphorylation and dephosphorylation, respectively. The effects of the FE65 mutants on γ-secretase-mediated APP processing were monitored by the APP-GAL4 cleavage reporter assay. As shown in Fig. 3A, overexpression of both FE65 and T579A dephosphomimetic mutant promoted the γ-secretase cleavage of APP to a similar extent. Remarkably, FE65 T579E phosphomimetic mutant had a significantly stronger effect that the wild-type counterpart (Fig. 3A).

Next, we sought to determine if FE65 T579 phosphorylation influences β-secretase (BACE1) cleavage of APP by monitoring APP CTF-β and -β’ production. Cells were transfected with APP + BACE1 + FE65 wild-type/T579A/T579E, and the cleavage patterns of APP were determined by immunoblotting for CTFs. As shown in Fig. 3B, a strong APP CTF-α and a faint CTF-β’ bands were observed in APP singly transfected cells. Akin to other reports^9,12,15–17^, the levels of CTF-β and -β’ were markedly increased when BACE1 was co-transfected. Similar to the APP-GAL4 cleavage reporter assay, both FE65 and T579A dephosphomimetic mutant enhanced the production of CTF-β and -β’. Notably, more CTF-β and -β’ were observed in cells co-transfected with FE65 T579E phosphomimetic mutant (Fig. 3B) than wild-type and T579A dephosphomimetic mutant.

Consistent with the above two assays, we also found that FE65 T579E phosphomimetic mutation potentiated the effect of FE65 on both Aβ1–40 and 1–42 liberation (Fig. 3C). Taken together, our findings suggest that FE65 T579 phosphorylation promotes the amyloidogenic processing of APP, which in turn potentiates the liberation of Aβ.

### FE65 T579 phosphorylation suppresses FE65-PTB2 dimerization and enhances FE65/APP interaction

**Figure 4:**
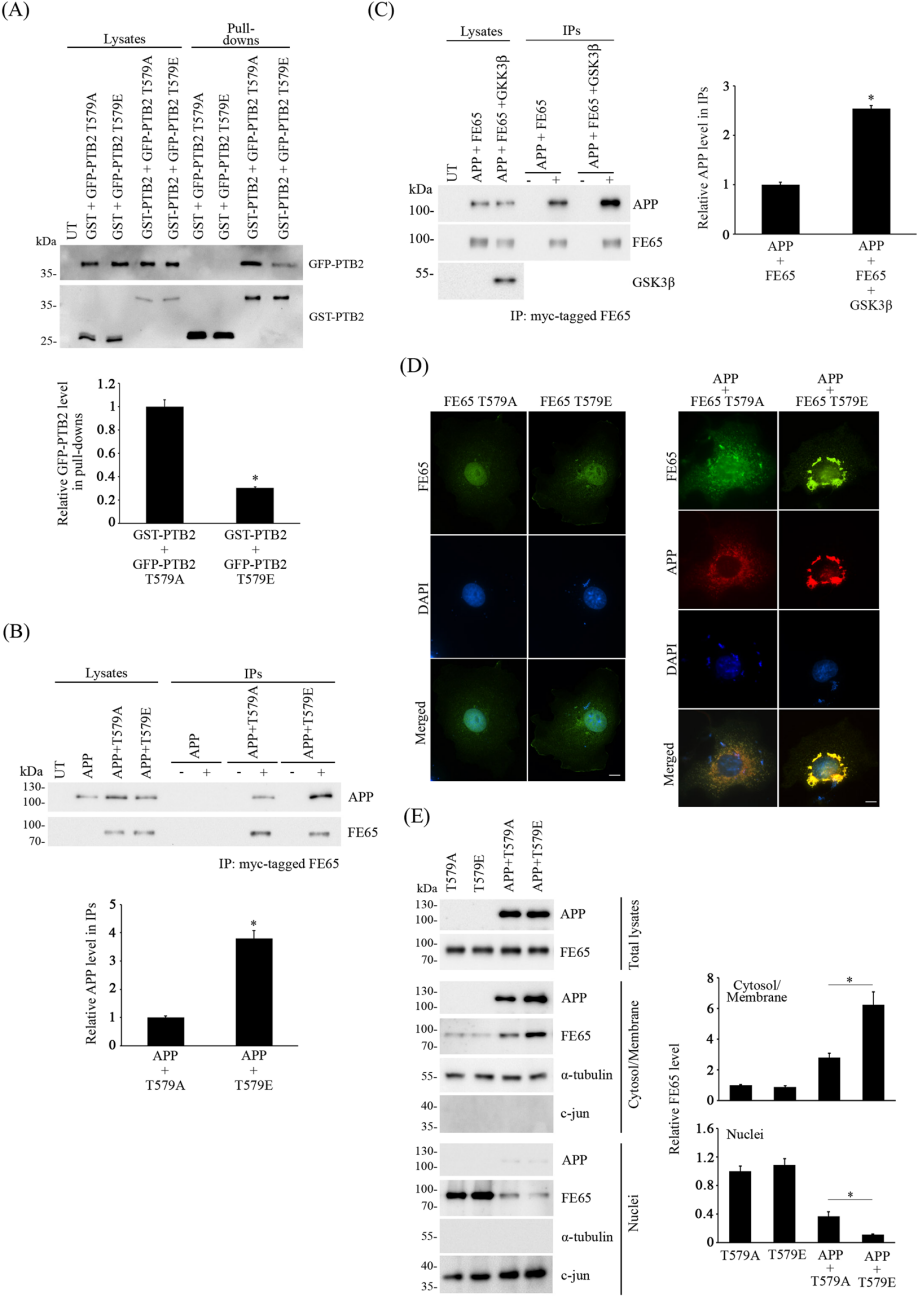
FE65 T579 phosphorylation enhances FE65/APP interaction but suppresses FE65-PTB2 dimerization. (**A**) Cells were transfected with GST + FE65 PTB2 T579A or T579E and GST-FE65 PTB2 + GFP-FE65 PTB2 T579A or T579E. GST and GST-FE65 PTB2 were pulled down from the cell lysates. Levels of GST or GST-FE65 PTB2 and GFP-FE65 PTB2 present in cell lysates and pull-downs were determined with anti-GST and anti-GFP antibodies respectively. The relative amounts of GFP-PTB2 in the immunoprecipitates were analyzed by densitometry. n = 3. *P < 0.001. Results are means ± S.D. UT, untransfected. CHO cells were transfected with (**B**) APP and APP + FE65 T579A or T579E (**C**) APP + FE65 or APP + FE65 + GSK3β. FE65 was immunoprecipiated by anti-myc 9B11 antibody to its myc-tag. Levels of APP, FE65 and GSK3β present in cell lysates and immunocomplex were determined using anti-APP, anti-FE65 and anti-HA antibodies respectively. The relative amounts of APP level in the immunoprecipitates were analyzed by densitometry. n = 3. *P < 0.001. Results are means ± S.D. For (**B**) and (**C**), 40% of cell lysate was used for each immunoprecipitation, and + and - denotes the presence or absence of anti-myc antibody in the immunoprecipitation. 5% of lysates were loaded for both (**B**) and (**C**). UT, untransfected. IPs, immunoprecipitations. (**D**) Immunofluorescence staining of COS-7 cells with FE65 T579A, FE65 T579E, APP + FE65 T579A and APP + FE65 T579E. Nucleus were stained by DAPI. Scale bars are 10μm. Representative line scan traces across an APP + FE65 T579A cell and an APP + FE65 T579E cell are shown. (**E**) Subcellular fractionation of FE65 T579A, FE65 T579E, APP + FE65 T579A and APP + FE65 T579E from transfected CHO cells. The total lysates, cytosol/membrane fractions and nuclear fractions were probed with antibodies for APP and FE65. The purity of the fractions were revealed by probing with anti α-tubulin (cytosol/membrane) and c-jun (nuclei). The relative FE65 level in the cytosol/membrane fraction and nuclear fraction were analyzed by densitometry. n = 3. *P < 0.001. Results are means ± S.D.

Previous finding suggests that FE65 PTB2 phosphorylation could modulate APP processing by altering FE65/APP interaction^9^. However, the crystal structure of FE65 PTB2/AICD complex (AICD-PTB2) indicates that FE65 T579 is situated on the opposite side of FE65/APP interaction interface^18^. Therefore, it is possible that FE65 T579 phosphorylation regulates APP processing by a mechanism other than direct alteration of FE65/APP interaction. A recent study suggests that FE65 forms intermolecular homodimer in cells via the PTB2 domain, and such dimerization shields the hydrophobic pocket where APP binds PTB2 domain^19^. Therefore, we enquired if FE65 T579 phosphorylation influences such dimerization as it could affect the availability of monomeric FE65 for binding to APP. To do this, we transfected cells with GST or GST-FE65 PTB2 + GFP-FE65 PTB2 T579A or T579E, and then pulled down GST and GST-FE65 PTB2 from the cell lysates by using glutathione-Sepharose 4B. As shown in Fig. 4A, GST-FE65 PTB2 pulled down less FE65 PTB2 T579E than T579A mutant suggesting that FE65 T579 phosphorylation interferes FE65 dimerization. GFP-PTB2 was not detected in the GST + GFP-FE65 PTB2 T579A or T579E control pull-downs (Fig. 4A).

As stated above, the level of monomeric FE65 could alter cellular FE65/APP complex formation. Therefore, we investigated if the FE65 T579E phosphomimetic mutation affects the formation of FE65/APP complex. To test this, APP was co-transfected with either myc-tagged FE65 T579A or T579E for co-immunoprecipitation assays. FE65 T579A and T579E were immunoprecipitated from the cell lysates by anti-myc antibody, and the amount of APP in the immunocomplexes were determined by immunoblotting. Despite similar amounts of FE65 T579A and T579E were immunoprecipiated, more APP was detected in the APP + FE65 T579E immunoprecipitate (Fig. 4B). This finding suggests that more APP is complexed with FE65 T579 phosphomimetic mutant than the dephosphomimetic counterpart. Similarly, the amount of FE65/APP complex was markedly increased in the presence of GSK3β in co-immunoprecipitation assays (Fig. 4C).

It has been proposed that APP serves as a cytosolic tethering site for FE65 to prevent FE65 nuclear localization^20^. Since FE65 T579 phosphorylation enhances FE65/APP interaction by disrupting FE65 dimerization, we enquired if such phosphorylation affects FE65 subcellular localization by phosphomimetic approach. FE65 T579A and T579E were transfected to COS7 cells either alone or with APP. As shown in Fig. 4D (left panel), both FE65 T579A and T579E were mainly observed in the nuclei. When co-transfected with APP, APP and FE65 T579A co-localized in the perinuclear region (Fig. 4D, right panel) which is consistent with the finding that APP retains FE65 in cytoplasm^20^. On the other hand, more intense APP and FE65 co-localization staining was observed in the perinuclear region of APP + FE65 T579E transfected cells (Fig. 4D, right panel). To confirm the above observation, we prepared nuclei and cytosol/membrane fractions from transfected cells. Similar levels of FE65 T579A and T579E were observed in both nuclei and cytosol/membrane fractions from singly transfected cells (Fig. 4E). Intriguingly, upon co-transfection with APP, the level of FE65 T579E was increased in the cytosol/membrane fraction, but reduces in nuclei fraction, when compare with the T579A dephosphomimetic mutant (Fig. 4E). These findings may suggest that FE65 T579 phosphorylation promotes FE65 cytoplasmic retention by enhancing FE65/APP interaction. Nevertheless, our data suggests that FE65 T579 phosphorylation enhances FE65/APP complex formation by interfering FE65 homodimerization.

## Discussion

Aberrant protein phosphorylation is associated with AD, and GSK3β has been strongly implicated in the disease pathogenesis in particular for its role in hyperphosphorylation of microtubule associated protein tau. On the other hand, several studies indicate that GSK3β activity is also associated with increased Aβ generation. For instance, suppression of GSK3β reduces Aβ toxicity in both *Drosophila* and mouse models of AD^21,22^. However, the mechanism(s) by which GSK3β exerts its effect(s) on APP processing is still not fully understood. FE65 is a phosphoprotein that has been shown to interact with GSK3β^23,24^, but whether FE65 is a substrate for GSK3β and the effect of such FE65/GSK3β interaction on APP processing were not known. In this study, we showed that FE65 T579 is phosphorylated by GSK3β by various assays. Importantly, FE65 T579 phosphorylation is found to potentiate FE65/APP interaction, a process that is reported to be crucial for Aβ generation^25^. In fact, hereby we demonstrated that FE65 T579 phosphorylation enhances APP processing and Aβ production.

The exact mechanism(s) by which FE65 modulates APP processing remains elusive. In addition to APP, FE65 interacts with LRP1 via its PTB1 domain. It is proposed that LRP1/FE65/APP promotes amyloidogenic processing of APP by increasing endocytosis that makes APP more accessible to the endosomal BACE1 and γ-secretase^26^. The availability of FE65 for recruiting LRP1 and APP may be crucial for the formation of the trimeric complex. FE65 molecules have been shown to form homodimer via their PTB2 domains which prevents the domain from binding to APP^19^. Such dimerization of FE65 PTB2 domains may serve as a mechanism to regulate the availability of monomeric FE65 for binding to APP. Noteworthy, the dimerization involves APP binding pocket formed by PTB2 helix α3 and β5 and FE65 c-terminal β strand (β^ct^), which is formed from the dissolved helical turns at the c-terminus^19^ (Fig. 1A). Since FE65 T579 is located in PTB2 β2 strand which does not make any direct contact with the dimerization regions^18^, it is not known how FE65 T579 phosphorylation facilitates FE65-dimer dissociation. It is possible that the phosphorylation induces allosteric conformational change of FE65 PTB2, and such mechanism is observed in some phosphoproteins. For example, phosphorylation at the c-terminus of Rap1b communicate allosterically with the distal N-terminal effector binding domain to modify effector protein interaction^27^. Nevertheless, our findings reveal that such dimerization of FE65 PTB2 could be disrupted by FE65 T579 phosphorylation by GSK3β. In this regards, GSK3β may promote amyloidogenic processing of APP by regulating the cellular level of monomeric FE65 for the formation of LRP1/FE65/APP complex.

Although many literatures suggest that FE65 promotes APP processing, contradictory findings are also reported^28–30^. The reason for such controversy is largely unknown. Here, we showed that FE65 T579 phosphorylation stimulates APP processing by potentiating FE65/APP interaction. In contrast, FE65 S610 phosphorylation has suppressive effect on both APP processing and FE65/APP interaction^9^. Therefore, the overall effect of FE65 on APP processing could be determined by the combined effect of different FE65 phosphorylated residues that influence FE65/APP interaction, not limited to FE65 T579 and S610. In addition to APP, FE65 phosphorylation has been shown to influence its interaction with other binding partners. For instance, FE65 Y547 phosphorylation reduces FE65/Dexras1 interaction to stimulate FE65-APP-mediated transcription^5^. Noteworthy, several FE65 interactors are shown to influence FE65-meidated APP metabolism. As stated before, FE65/LRP1 interaction is essential for the amyloidogenic processing of APP. Although the effect of FE65 phosphorylation on FE65/LRP1 interaction is not known, phosphorylation of LRP1 has been shown to alter FE65/LRP1 interaction^31^. It is therefore possible that the effect of FE65 on APP processing depends on the overall FE65 phosphorylation status which in turn alter its interaction with APP and/or other interactors. Therefore, identification of the full complement of FE65 kinases and their target residues in future studies will provide further insights into this issue.

In summary, we have identified GSK3β as a kinase for FE65 T579. Moreover, our findings suggest that FE65 T579 phosphorylation promotes FE65/APP complex formation and FE65-mediated APP processing by reducing FE65 PTB2 homodimerization.

## Methods

### Plasmids

Mammalian expression constructs for wild-type FE65, myc-tagged FE65, GST-FE65 PTB2 (531–676) and HA-tagged GSK3β were as described^32–35^. Phosphomimetic (T579E) and dephosphomimetic (T579A) mutants were generated by using QuikChange II site-directed mutagenesis kit (Agilent Technologies). The GFP-tagged constructs of FE65 PTB2 was generated by subcloning corresponding FE65 cDNA into pEGFP-N1, and T579 phosphomimetic and dephosphomimetic mutations were introduced by using QuikChange II site-directed mutagenesis kit. The pRcCMV GAL4-APP plasmid, which encodes for the chimeric APP695 with full-length GAL4 yeast transcription factor fused to C-terminus, was as described^36^. GAL4-dependent firefly luciferase reporter pFR-Luc and *Renilla* luciferase reporter phRL-TK were from Agilent Technologies and Promega, respectively.

### Cell culture and transfection

CHO and HEK293 were prepared and cultured as described previously^12,35^. DNA constructs were transfected to the cells by using X-tremeGENE 9 (Roche) according to the manufacturer's instruction.

### Antibodies

Rabbit anti-FE65 and rabbit anti-APP A5137 were as described^5^. Goat anti-FE65 E20 and mouse anti-α-tubulin DM1A were obtained from Santa Cruz. Mouse anti-myc 9B11, mouse anti-GFP JL8, mouse anti-HA 12CA5, rabbit anti-GST and mouse anti-APP 22C11 were purchased from Cell Signaling Technology, Clontech, Roche, Sigma and Millipore, respectively.

The phospho-specific antibody against phosphorylated FE65 at T579 (pT579 FE65) was generated by immunizing rats with a synthetic FE65 phosphopeptide (amino acids 575–586; REQW(pT)PSHVSVC) (GenScript) which contains a phosphorylated T579. A cysteine (C) residue was introduced to the peptide C-terminus for carrier protein conjugation and purification column coupling. Phosphopeptide conjugation was performed using the Imject Maleimide Activated mcKLH Spin kit (ThermoFisher Scientific). The immunized rat sera were purified by the non-phosphopeptide (REQWTPSHVSVC) and then phosphopeptide coupled columns prepared by using a SulfoLink Immobilization kit for peptides (ThermoFisher Scientific). The antibody was eluted and dialyzed against PBS/0.05% sodium azide, and then concentrated by using Amicon Ultra-15 Centrifugal Filter Unit with Ultracel-10 membrane (Millipore).

### Protein binding assays

Co-immunoprecipitation assays were performed essentially as described previously from transfected cell and rat brain lysates^5,12,35^.

For FE65 PTB2 dimerization assay, CHO cells were transfected with GST + GFP-FE65 PTB2 T579A or T579E and GST-FE65 PTB2 + GFP-FE65 PTB2 T579A or T579E. The cells were harvested in ice-cold cell lysis buffer as described previously^9^ and cleared by centrifugation at 4 °C for 10 mins at 15,000 rpm. GST and GST-FE65 PTB2 were pulled down from the lysates by incubating with Glutathione Sepharose 4B (GE Healthcare) at 4 °C for 4 hours. The pull-downs were analysed for the presence of GST-FE65 PTB2 and GFP-FE65 PTB2 by immunoblotting. Signals on the immunoblots were captured by using a C-DiGit® Blot Scanner (LI-COR). Densitometry was performed by using LI-COR® Image Studio™ Software.

For detection of T579 phosphorylated FE65, FE65 was immunoprecipitated from rat brain lysate by goat anti-FE65 E20. FE65 and T579 phosphorylated FE65 in the immunoprecipiate were detected by rabbit anti-FE65 and pT579 FE65, respectively.

### *In vitro* kinase assays

*In vitro* kinase assays were performed as described previously^9^. Wild-type and T579A His-tagged FE65 PTB2 (residues 531 to 666) recombinant proteins were expressed and purified from *E. coli* as substrates. HA-GSKβ was isolated from transfected cell lysate by immunoprecipitation using anti-HA 12CA5 antibody (Roche).

### APP processing assays

APP-GAL4 luciferase reporter assay, Tris-Tricine SDS-PAGE analysis for APP CTFs and Aβ ELISA assay were performed as described previously^9,12^.

### Indirect immunofluorescence

Transfected COS-7 cells grown on glass coverslips were fixed and processed as previously described^5^. APP and FE65 were detected with a mouse anti-APP antibody (22C11) and a rabbit anti-FE65 antibody^5^, respectively. The primary antibodies were visualized by Alexa Fluor® 488 and 594 secondary antibodies (Invitrogen). 4′,6-diamidino-2-phenylindole (DAPI) was purchased from Sigma for nuclei staining. Images were captured with an inverted fluorescence microscope equipped with a Nikon DS-Qi2 camera.

### Subcelluar Fractionation

Subcelluar fractionation of cells transfected with APP and FE65 was performed as described^5,37^. The purity of the different fractions were determined by probing with fraction-specific markers (α-tubulin and c-Jun).

### Statistical analysis

One-way Analysis of Variance (ANOVA) with Turkey-Kramer Multiple Comparisons Test was performed for APP-GAL4 reporter and Aβ ELISA assays. Differences were considered statistically significant if the *P*-value is less than 0.001. Significance is indicated as *P < 0.001. Error bars were shown as standard deviation.

## Electronic supplementary material

Supplementary data

## References

[1] O’Brien RJ, Wong PC (2011). Amyloid precursor protein processing and Alzheimer’s disease. Annu Rev Neurosci.

[2] McLoughlin DM, Miller CC (2008). The FE65 proteins and Alzheimer’s disease. J Neurosci Res.

[3] Borquez DA, Gonzalez-Billault C (2012). The amyloid precursor protein intracellular domain-fe65 multiprotein complexes: a challenge to the amyloid hypothesis for Alzheimer’s disease?. Int J Alzheimers Dis.

[4] Lee EJ (2008). Regulation Fe65 localization to the nucleus by SGK1 phosphorylation of its Ser566 residue. BMB Rep.

[5] Lau KF (2008). Dexras1 Interacts with FE65 to Regulate FE65-Amyloid Precursor Protein-dependent Transcription. J Biol Chem.

[6] Standen CL (2003). The neuronal adaptor protein Fe65 is phosphorylated by mitogen-activated protein kinase (ERK1/2). Mol Cell Neurosci.

[7] Xue Y (2008). GPS 2.0, a tool to predict kinase-specific phosphorylation sites in hierarchy. Mol Cell Proteomics.

[8] Horn H (2014). KinomeXplorer: an integrated platform for kinome biology studies. Nat Methods.

[9] Chow WN (2015). Phosphorylation of FE65 Serine-610 by serum- and glucocorticoid-induced kinase 1 modulates Alzheimer’s disease amyloid precursor protein processing. Biochem J.

[10] Langlands H, Blain PG, Jowsey PA (2016). Fe65 Is Phosphorylated on Ser289 after UV-Induced DNA Damage. PLoS One.

[11] Jowsey PA, Blain PG (2015). Fe65 Ser228 is phosphorylated by ATM/ATR and inhibits Fe65-APP-mediated gene transcription. Biochem J.

[12] Hao CY (2011). GULP1 is a novel APP-interacting protein that alters APP processing. Biochem J.

[13] Minopoli G, Gargiulo A, Parisi S, Russo T (2012). Fe65 matters: New light on an old molecule. IUBMB Life.

[14] Sabo SL (1999). Regulation of b-amyloid secretion by FE65, an amyloid protein precursor-binding protein. J Biol Chem.

[15] Repetto E (2004). BACE1 overexpression regulates amyloid precursor protein cleavage and interaction with the ShcA adapter. Ann N Y Acad Sci.

[16] Sala Frigerio C (2010). beta-Secretase cleavage is not required for generation of the intracellular C-terminal domain of the amyloid precursor family of proteins. FEBS J.

[17] Vetrivel KS (2011). Loss of cleavage at beta’-site contributes to apparent increase in beta-amyloid peptide (Abeta) secretion by beta-secretase (BACE1)-glycosylphosphatidylinositol (GPI) processing of amyloid precursor protein. J Biol Chem.

[18] Radzimanowski J (2008). Structure of the intracellular domain of the amyloid precursor protein in complex with Fe65-PTB2. EMBO Rep.

[19] Feilen LP (2017). Fe65-PTB2 Dimerization Mimics Fe65-APP Interaction. . Front Mol Neurosci.

[20] Minopoli G (2001). The b-amyloid precursor protein functions as a cytosolic anchoring site that prevents Fe65 nuclear translocation. J Biol Chem.

[21] Sofola O (2010). Inhibition of GSK-3 ameliorates Abeta pathology in an adult-onset Drosophila model of Alzheimer’s disease. PLoS Genet.

[22] Ly PT (2013). Inhibition of GSK3beta-mediated BACE1 expression reduces Alzheimer-associated phenotypes. J Clin Invest.

[23] Chow WN, Cheung HN, Li W, Lau KF (2015). FE65: Roles beyond amyloid precursor protein processing. Cell Mol Biol Lett.

[24] Lee EJ (2008). The PPLA motif of glycogen synthase kinase 3beta is required for interaction with Fe65. Mol Cells.

[25] Barbagallo AP (2010). Tyr(682) in the intracellular domain of APP regulates amyloidogenic APP processing *in vivo*. PLoS One.

[26] Pohlkamp T, Wasser CR, Herz J (2017). Functional Roles of the Interaction of APP and Lipoprotein Receptors. Front Mol Neurosci.

[27] Edreira MM (2009). Phosphorylation-induced conformational changes in Rap1b: allosteric effects on switch domains and effector loop. J Biol Chem.

[28] Ando K, Iijima KI, Elliott JI, Kirino Y, Suzuki T (2001). Phosphorylation-dependent regulation of the interaction of amyloid precursor protein with Fe65 affects the production of b-amyloid. J Biol Chem.

[29] Hoe HS (2006). FE65 interaction with the ApoE receptor ApoEr2. J Biol Chem.

[30] Santiard-Baron D (2005). Expression of human FE65 in amyloid precursor protein transgenic mice is associated with a reduction in b-amyloid load. J Neurochem.

[31] Klug W, Dietl A, Simon B, Sinning I, Wild K (2011). Phosphorylation of LRP1 regulates the interaction with Fe65. FEBS Lett.

[32] Lau KF, McLoughlin DM, Standen CL, Irving NG, Miller CC (2000). Fe65 and X11b co-localize with and compete for binding to the amyloid precursor protein. Neuroreport.

[33] Perkinton MS (2004). The c-Abl tyrosine kinase phosphorylates the Fe65 adaptor protein to stimulate Fe65/amyloid precursor protein nuclear signaling. J Biol Chem.

[34] Cao X, Sudhof TC (2001). A transcriptionally [correction of transcriptively] active complex of APP with Fe65 and histone acetyltransferase Tip60. Science.

[35] Cheung HN (2014). FE65 interacts with ADP-ribosylation factor 6 to promote neurite outgrowth. FASEB J.

[36] Zambrano N (2004). Fe65 is not involved in the platelet-derived growth factor-induced processing of Alzheimer’s amyloid precursor protein, which activates its caspase-directed cleavage. J Biol Chem.

[37] Thomas JE (1996). Subcellular localization and analysis of apparent 180-kDa and 220-kDa proteins of the breast cancer susceptibility gene, BRCA1. J Biol Chem.

