# Supplementary material for: Phosphorylation of FE65 at threonine 579 by GSK3β stimulates amyloid precursor protein processing
Source: Sci Rep. 2017 Sep 29;7:12456. doi: 10.1038/s41598-017-12334-2 (PMC5622059; doi:10.1038/s41598-017-12334-2)

Phosphorylation of FE65 at threonine 579 by GSK3β stimulates amyloid precursor protein processing

Yat Shing Lee[1], Wan Ning Vanessa Chow[1] and Kwok-Fai Lau[1*]

[1]School of Life Sciences, Faculty of Science, The Chinese University of Hong Kong, Shatin, N.T., Hong Kong SAR

[*] Corresponding author: kflau@cuhk.edu.hk (email)

Figure 1 full length blots

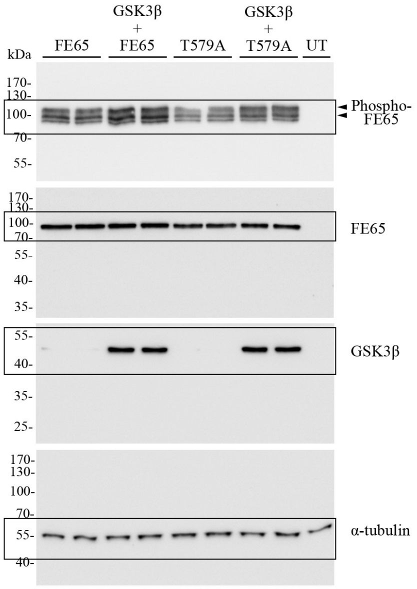

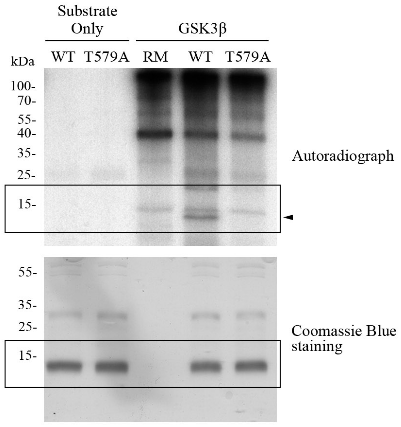

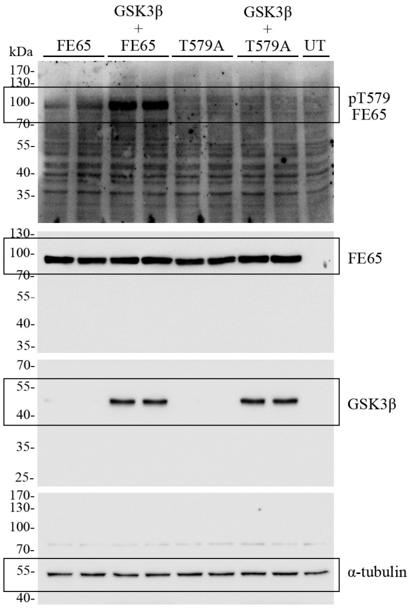

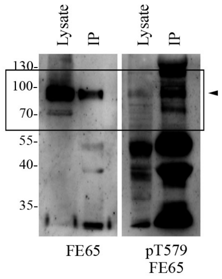

Figure 3 full length blots

(A)

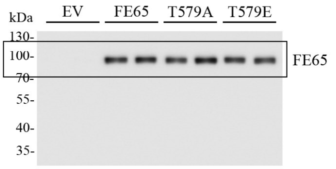

(C)

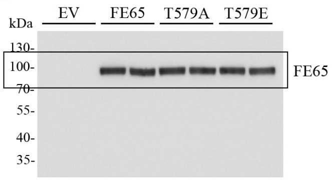

(B)

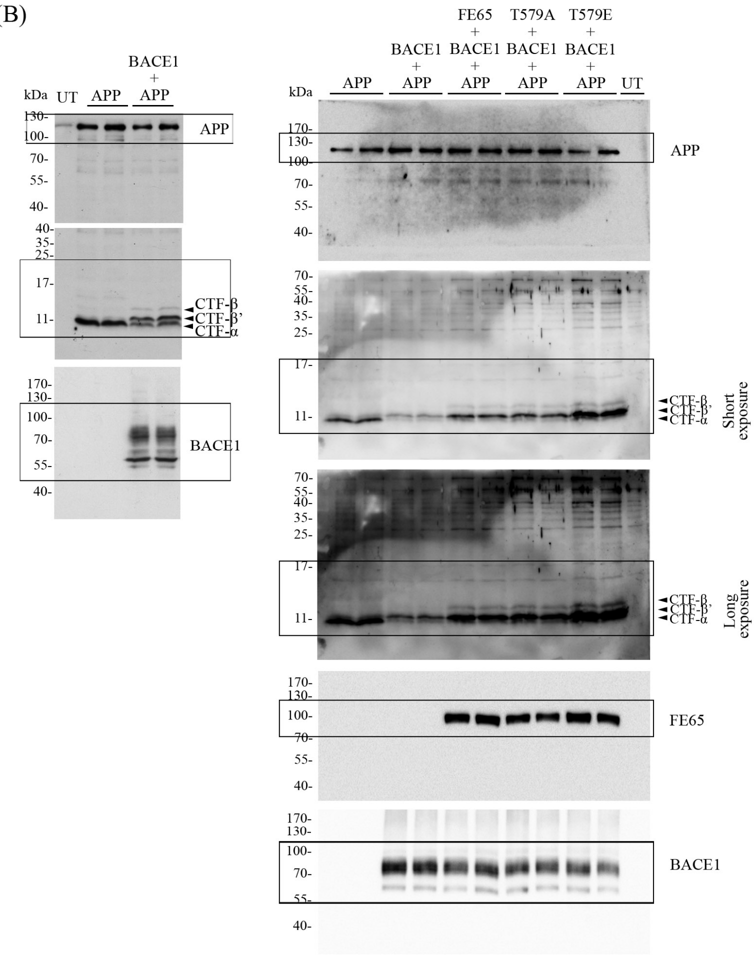

Figure 4 full length blots

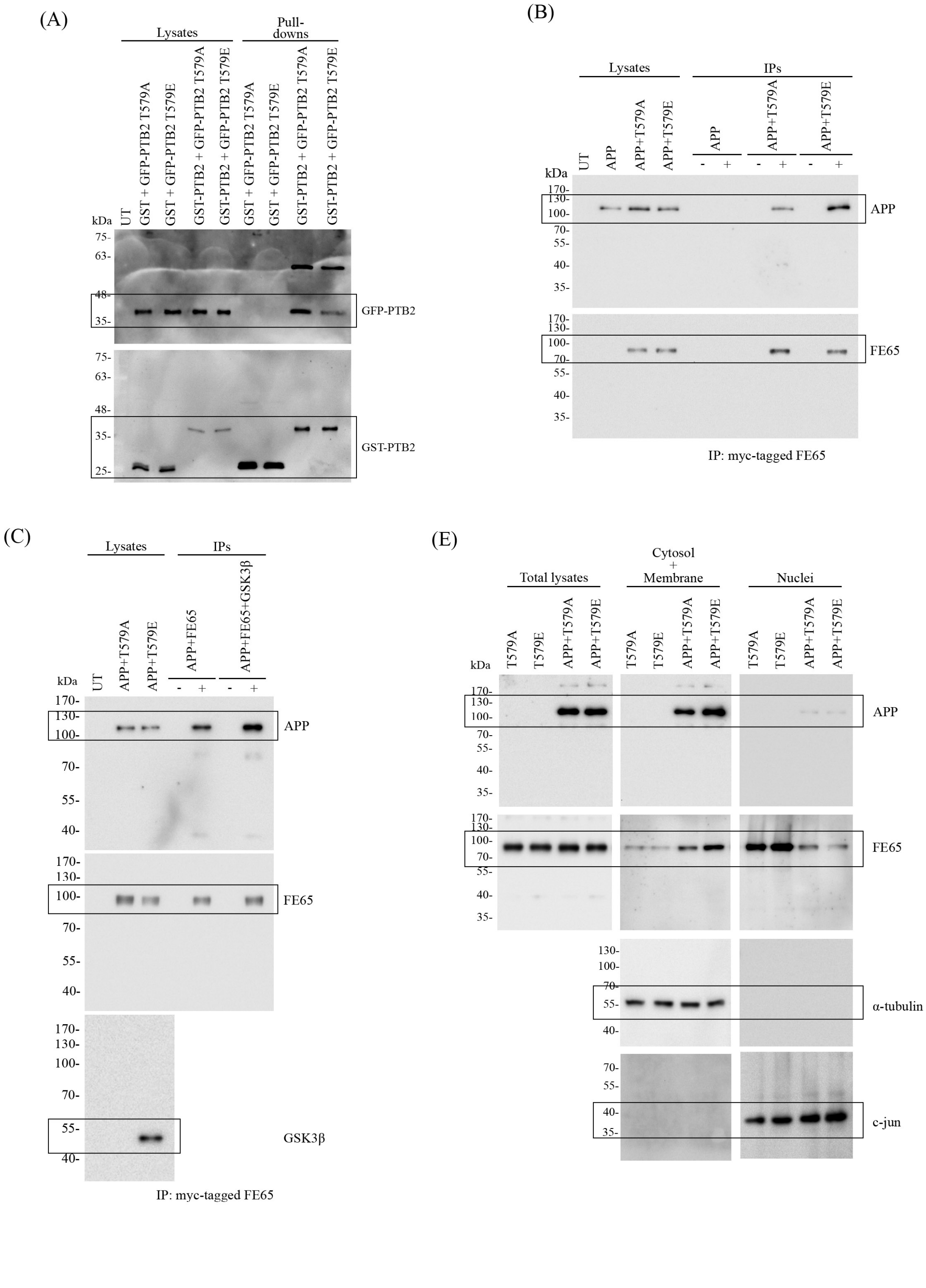

Supplement: Supplementary file 1 — Supplementary data [file 41598_2017_12334_MOESM1_ESM.pdf]
